# Low GAS5 Levels as a Predictor of Poor Survival in Patients with Lower-Grade Gliomas

**DOI:** 10.1155/2019/1785042

**Published:** 2019-02-03

**Authors:** Yanfang Wang, Shan Xin, Kai Zhang, Run Shi, Xuanwen Bao

**Affiliations:** ^1^Ludwig-Maximilians-Universität München (LMU), 80539 Munich, Germany; ^2^Department of Cardiology, Sir Run Run Shaw Hospital, Zhejiang University School of Medicine, 310016 Hangzhou, China; ^3^Institute of Radiation Biology, Helmholtz Center Munich, German Research Center for Environmental Health, 85764 Neuherberg, Germany; ^4^Technical University Munich (TUM), 80333 Munich, Germany

## Abstract

**Introduction:**

Gliomas are infiltrative neoplasms of a highly invasive nature. Different stages of gliomas feature distinct genomic, genetic, and epigenetic changes. The long noncoding RNA Growth Arrest Specific Transcript 5 (GAS5) is an identified tumour suppressor involved in several cancers. However, the underlying roles of the GAS5 gene in lower-grade glioma (LGG) patients are not clear.

**Methods:**

Via bioinformatic analysis based on TCGA-LGG and TCGA-GBM data, we explored the mechanisms of GAS5 expression in LGG (grades II and III) and high-grade glioma (glioblastoma multiforme, grade IV). The log-rank test and multivariate Cox analysis were performed to find the association between GAS5 and overall survival (OS) in LGG patients. Weighted gene coexpression network analysis (WGCNA) and RNA-Seq analysis were applied to find the key gene network associated with GAS5.

**Results:**

We found that GAS5 expression was downregulated in both LGG and glioblastoma multiforme (GBM) compared with normal brain tissue. Low methylation in the GAS5 promoter region was detected in both LGG and GBM tissues. The amplification type was the predominant type of GAS5 gene alteration in both LGG and GBM. High GAS5 expression was more associated with long overall survival (OS) in LGG patients than in GBM patients. The multivariate survival analysis of GAS5 and clinical and molecular characteristics in LGG patients further confirmed the association between GAS5 and OS in LGG patients. We then developed a nomogram for clinical use. WGCNA and RNA-Seq analysis indicated that ribosomal biogenesis and translation initiation were the predominant events regulated by GAS5 in LGG patients.

**Conclusion:**

Taken together, these results demonstrate that GAS5 expression is associated with OS in LGG patients and that its underlying roles involve the regulation of ribosomal biogenesis and translation initiation, which may aid in identifying a new target for the treatment of LGG.

## 1. Introduction

Gliomas are commonly classified as low-grade glioma (LGG, grades II and III) or glioblastoma multiforme (GBM, grade IV). LGGs are infiltrative neoplasms that arise most often in the cerebral hemispheres of adults, including oligodendrogliomas, oligoastrocytomas, and astrocytomas [1]. A subset of LGGs progress into GBM within a few months, and others may stay stable for several years, causing large variations in median survival [2, 3]. Hence, individual treatments should be performed based on the identification of histologic class, grade, and reactions to chemotherapy and radiotherapy [4]. GBM is the most common cause of death among children with central nervous system (CNS) neoplasms, and no effective therapies currently exist [5]. In most cases, GBM will recur after surgical resection, resulting in a poor prognosis. Hundreds of molecular alterations exist in LGG and GBM, making them react differently to chemotherapy, radiotherapy, and surgical resection. Identifying the key genes or genome alterations that drive the progression of gliomas will contribute to the understanding of the molecular mechanism behind gliomas and help to improve the effects of therapy.

Long noncoding RNAs (lncRNAs) are non-protein-coding transcripts that play essential roles in cellular regulation at various levels and in diverse biological functions, including chromatin modification, transcriptional regulation, cell differentiation, immune responses, and epigenetic regulation [6–8]. GAS5 produces a spliced lncRNA, which sensitizes cells to apoptosis by suppressing glucocorticoid-mediated induction of several responsive genes [9]. By binding to the DNA-binding domain of the glucocorticoid receptor, GAS5 acts as a decoy glucocorticoid response element (GRE), thus competing with DNA GREs to bind to the glucocorticoid receptor [9]. By analysing GAS5 level and clinical parameters, it has been found that GAS5 correlates with tumour progression and poor prognosis in many tumour types [10–13]. Some publications have also indicated that GAS5 is a predictor of survival in GBM patients [14, 15]. One study revealed an association between serum GAS5 level and the OS of GBM patients [14]. However, the potential roles of GAS5 involvement in gliomas and the regulatory mechanism of GAS5 expression in gliomas are not clearly identified. Besides, the difference of GAS5 expression and the regulatory mechanism of GAS5 expression in LGG and GBM patients have not been shown yet. In this study, via bioinformatic analysis, we explored the regulatory mechanisms of GAS5 expression in gliomas and compared its prognostic value in LGG and GBM. In addition, based on TCGA-LGG RNA-Seq datasets, we also applied WGCNA and enrichment analysis to identify the pathways highly associated with GAS5 in LGG and the network of genes highly associated with GAS5.

## 2. Materials and Methods

### 2.1. Clinical and Omic Data Download

RNA-Seq and DNA methylation datasets from patients with primary LGG or GBM were downloaded from the UCSC Xena browser (https://xenabrowser.net/) [16]. GAS5 mRNA expressions in different types of solid tumours and in corresponding normal tissues were obtained from TCGA datasets and GTEx datasets [17]. A box plot was created using ggplot2 [18].

### 2.2. WGCNA of RNA-Seq Data from LGG Patients and Network Visualization

The WGCNA R package was used to evaluate the correlation of GAS5 expression and module membership by the ‘p.weighted' function [19]. An adjacency matrix was generated to evaluate the weighted coexpression values between all the subjects in the probe set. The coexpression similarity *s*_*i*,*j*_ was defined as the absolute value of the correlation coefficient between the profiles of nodes *i* and *j*:

(1) $$\begin{array}{c} s_{i,j}=\left|\;cor\left(\;x_{i},x_{j}\;\right)\;\right| \end{array}$$

where *x*_*i*_ and *x*_*j*_ were expression value of for genes *i* and *j* and *s*_*i*,*j*_ represented Pearson's correlation coefficient of gene *i* and gene *j*.

A weighed network adjacency was defined by raising the coexpression similarity to a power *β*:

(2) $$\begin{array}{c} a_{i,j}=s_{i,j}^{\beta } \end{array}$$

with *β* ≥ 1 [20]. We selected the power of *β* = 4 and scale free R^2^ = 0.95 as the soft-thresholding parameter to ensure a signed scale-free coexpression gene network. The salmon and blue modules, which had most significant adjusted p-values, were selected. Genes involved in the salmon and blue modules and their weights as calculated by WGCNA were visualized by Cytoscape 3.4.0 [21]. GO analysis was performed using the clusterProfiler package [22] and Metascape (http://metascape.org/gp/index.html) [23]. Gene set enrichment analysis (GSEA) was applied by the GSEA software from Broad Institute [24].

### 2.3. Statistical Analysis

Statistical analysis was performed using R (R: A language and environment for statistical computing. R Foundation for Statistical Computing, Vienna, Austria. URL http://www.R-project.org/). The association between GAS5 expression and the clinicopathological features was evaluated by the different mathematical methods listed in the table [25]. The log-rank test was performed to assess the difference between the Kaplan-Meier curves. The fold change and Q value for samples with high and low GAS5 expression were calculated using the limma package [26]. A Q value < 0.05 was considered statistically significant.

## 3. Results

### 3.1. GAS5 Was Upregulated in Both LGG and GBM Compared with Normal Brain Tissues

**Figure 1:**
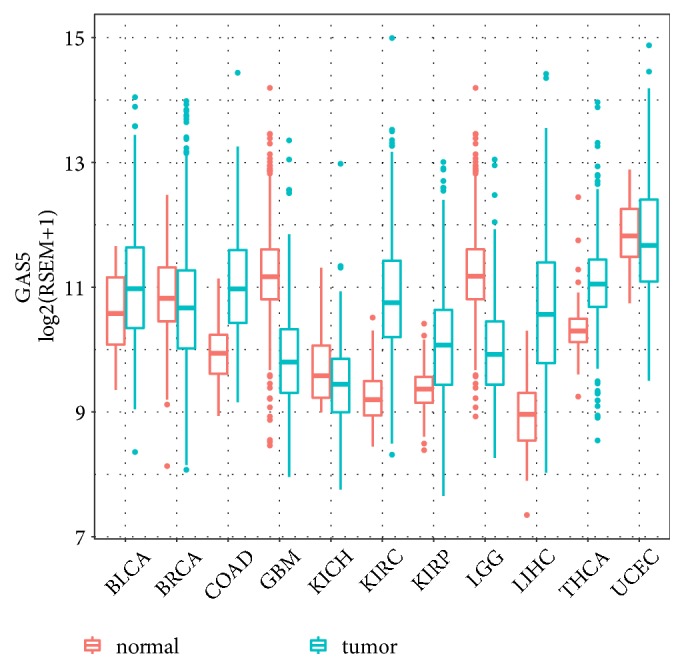
GAS5 mRNA expression in different types of solid tumours and corresponding normal tissues. The RNA-Seq data of normal brain tissue were obtained from GTEx dataset.

**Figure 2:**
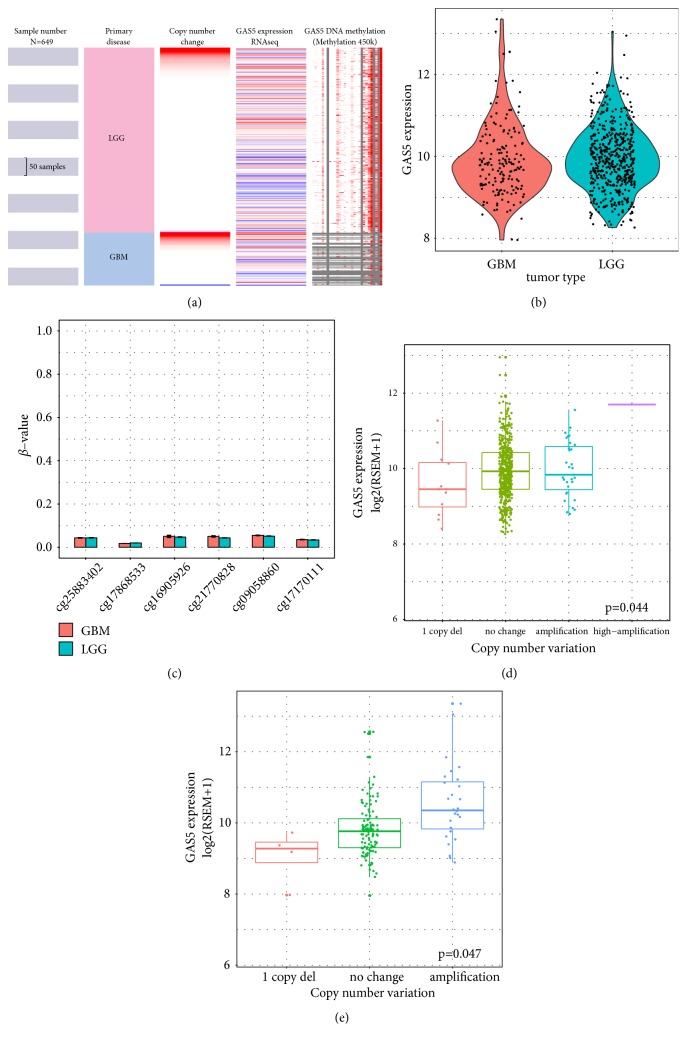
GAS5 expression, DNA methylation, and copy number alteration (CNA) in LGG and in GBM. (a) Heatmap of GAS5 mRNA expression, copy number alteration, and DNA methylation in patients with primary LGG or GBM. Data were obtained from TCGA-LGG and TCGA-GBM. (b) Violin plots of GAS5 expression in LGG and GBM tissues. (c) Bar plots of GAS5 methylation level in LGG and in GBM tissues; six probes were chosen from the CpG island before the transcription start site of GAS5. (d-e) Box plots of GAS5 expression in LGG (d) and GBM (e) tissues with indications of genetic status. Data were obtained from TCGA-LGG and TCGA-GBM.

GAS5 expression levels were characterized in several types of solid tumours, including LGG and GBM (Figure 1). The results indicated that GAS5 was downregulated in both LGG and GBM tissue compared with the normal brain tissue. Then, we compared the expression level of GAS5 between LGG and GBM (Figures 2(a), 2(b), and S1a). No significant difference was identified between the LGG and GBM datasets. In addition, grade II, grade III, and grade IV gliomas had similar expression levels of GAS5, which were all higher than that of normal brain tissues. The DNA methylation level of GAS5 was analysed by DNA methylation 450K chips, and the six probes were chosen from the CpG island in the promoter region of GAS5. All probes showed low *β* values (<0.1), and there were no differences in *β* values from any of the six probes between LGG and GBM tissues, indicating low methylation of the GAS5 promoter in both LGG and GBM patients (Figure 2(c)). The association between GAS5 methylation and GAS5 expression was tested, revealing no significant relationship in either GBM or LGG tissues (Figures S1B and S1C). Then, to investigate the driver of high GAS5 expression in LGG and GBM tissues, we also examined copy number alterations (CNAs) in LGG and GBM (Figures 2(d) and 2(e)). The results showed that the amplification-type alteration contributed to the high expression of GAS5 in both LGG and GBM tissues (P = 0.044 and P = 0.047).

### 3.2. Low GAS5 Expression Was a Predictor of Poor Survival in LGG

**Figure 3:**
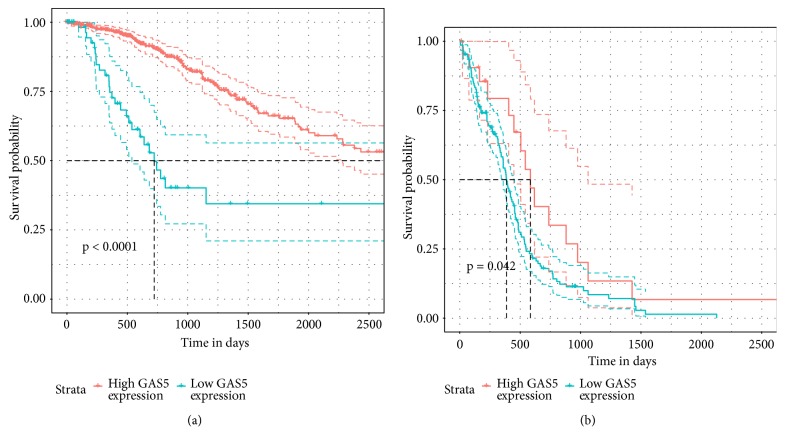
The associations between GAS5 expression and OS in LGG (a) and GBM (b) patients.

The associations between GAS5 expression and the pathological parameters of patients with primary LGG and GBM were summarized in Table 1. In patients with LGG, higher GAS5 expression was significantly correlated with higher tumour purity (Table 1 and Figure S2). Nevertheless, we could not find a similar tendency in patients with GBM. Furthermore, in LGG patients, higher expression of GAS5 was associated with longer OS according to the log-rank test (Figure 3(a)). However, the level of GAS5 expression did not show a strong association with the OS of GBM patients (Figure 3(b)).

### 3.3. GAS5 Expression in LGG Patients with Different Clinical and Molecular Characteristics

**Figure 4:**
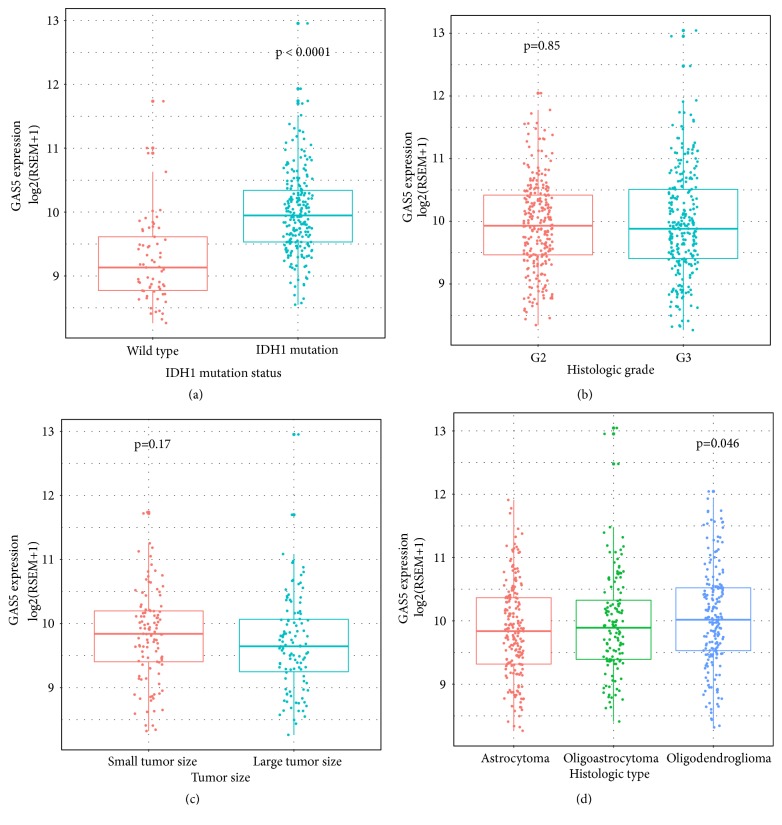
GAS5 expression in LGG patients with different IDH1 statuses (a); histologic grade (b); tumour sizes (c); and histological type (d).

**Figure 5:**
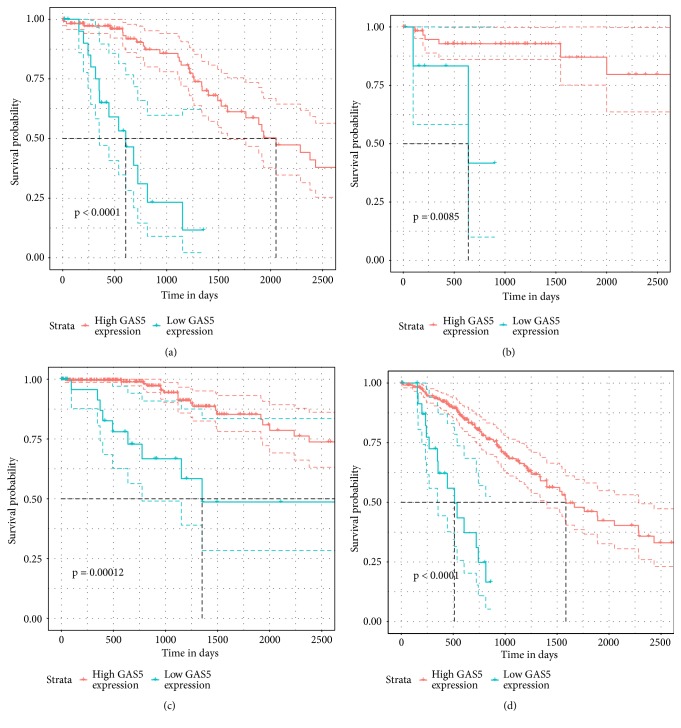
Kaplan-Meier survival analysis for LGG patients according to GAS5 expression levels, stratified by clinicopathological risk factors. (a, b) Tumour size. (c, d) Tumour grade. P values were calculated using the log-rank test.

To further identify the relationship between GAS5 expression and overall survival of LGG patients, we first examined GAS5 expression in LGG patients with different IDH1 status (Figure 4(a)), histologic grades (Figure 4(b)), tumour sizes (Figure 4(c)), and histologic types (Figure 4(d)). The results showed that the expression of GAS5 in IDH1 mutant patients was significantly higher than that in IDH1 wild-type patients. In contrast, histologic grade, tumour size, and histological type had no association with GAS5 expression. A Kaplan-Meier survival analysis stratified by clinicopathological risk factors was performed to verify the effect of GAS5 on survival. In patients with large or small tumours of histologic grade II or III, GAS5 showed a good predictive effect for survival (Figures 5(a)–5(d)).

### 3.4. Multivariate Survival Analysis of GAS5 with Clinical and Molecular Characteristics in LGG Patients

**Figure 6:**
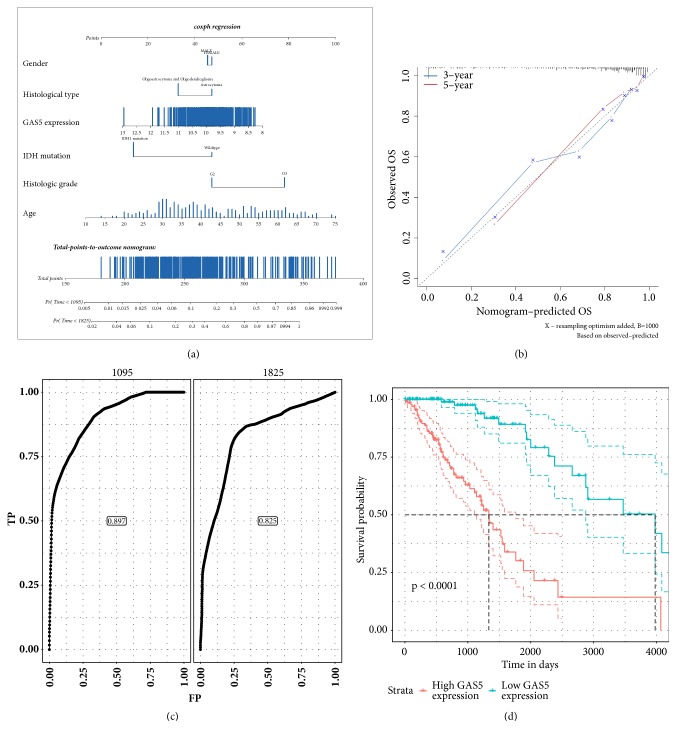
Multivariate survival analysis of GAS5 and clinical and molecular characteristics in LGG patients. Data are AUC (95% CI) or p values. ROC: receiver operating characteristic. AUC: area under the curve. (a) The nomogram to predict overall survival in LGG patients. (b) Plots depict the calibration of each model in terms of agreement between predicted and observed 3-year and 5-year outcomes. Model performance is shown by the plot, relative to the 45-degree line, which represents perfect prediction. (c) Time-dependent ROC curves indicate the prognostic accuracy of the nomogram over 3 and 5 years. We used AUCs at 3 and 5 years to assess prognostic accuracy. (d) Kaplan-Meier survival analysis was conducted for LGG patients in the high-risk-score group and the low-risk-score group from a Cox regression model, and p values were calculated using the log-rank test.

We applied the Cox regression model to conduct a multivariable survival analysis and used Cox regression coefficients to generate a nomogram (Figure 6(a)). In the multivariable survival analysis, we included histologic grade, gender, histological type, IDH mutation status, age, and GAS5 expression level as risk factors. The nomogram predicts LGG patients' overall 3-year and 5-year survival probability. The results indicated that the expression level of GAS5 was highly associated with overall survival in LGG patients. Calibration plots showed that the nomograms performed well compared with an ideal model (Figure 6(b)). Then, the risk scores from Cox regression were assessed with a time-dependent receiver operating characteristic (ROC) curve (Figure 6(c)). The area under the curve (AUC) was 0.897 and 0.825 for 3 years and 5 years, respectively. The patients were separated into 2 groups, namely, those with high and low risk scores according to the Cox regression model. The Kaplan–Meier curve, which was generated by the log-rank test, showed good performance in predicting the OS of LGG patients (Figure 6(d)).

### 3.5. WGCNA of RNA-Seq in LGG

**Figure 7:**
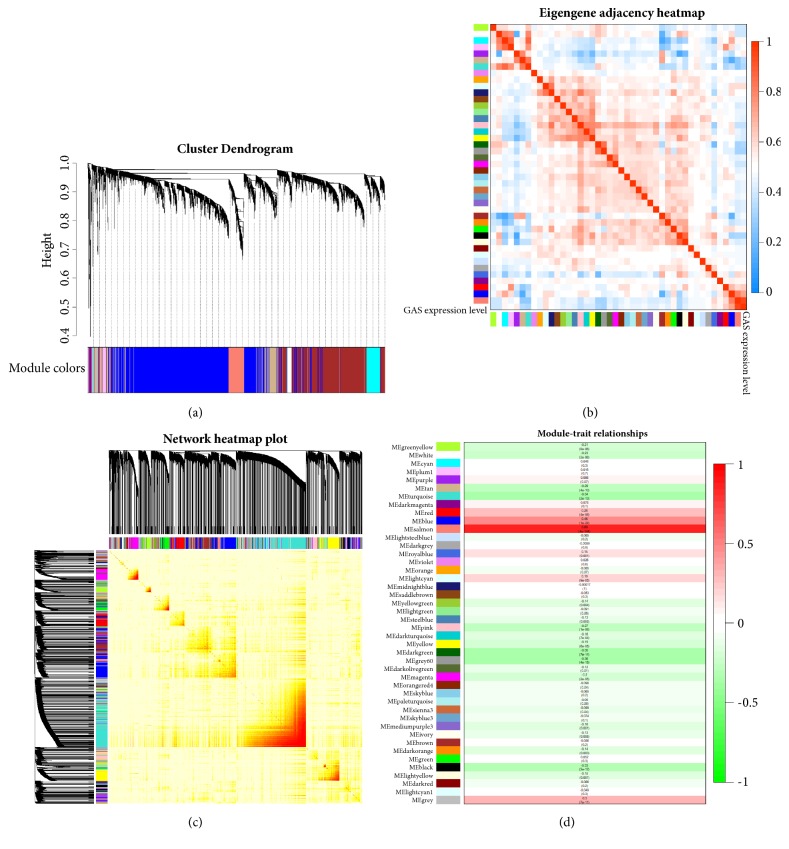
WGCNA of RNA-Seq data from LGG patients and GBM patients. (a) Dendrogram of modules identified by WGCNA. (b) Heatmap of module-GAS5 expression associations. (c) Topological overlap matrix among detected probes from RNA-Seq. (d) Heatmap for module similarity. (e-f) Scatterplot of gene significance for GAS5 expression versus module membership in the salmon (e) and blue modules (f).

To identify the gene network that was highly associated with GAS5 expression, we further analysed LGG RNA-Seq data by WGCNA. WGCNA has been widely applied to study coexpressed gene networks by scale-free weighted network construction and module detection. In our analysis, 43 modules were detected; the clustering of genes is shown as a dendrogram (Figures 7(a) and S3). The module similarity was quantified and is shown as a heatmap below (Figure 7(b)). The weighted network of identified genes from RNA-Seq is shown as a heatmap below, which depicts the topological overlap matrix among all genes (Figure 7(c)). Then, 26 modules were identified to be related with GAS5 expression by the heatmap of associations between modules and GAS5 expression (Figure 7(d)).

### 3.6. Enrichment Analysis of Genes Highly Associated with GAS5 Expression in LGG

**Figure 8:**
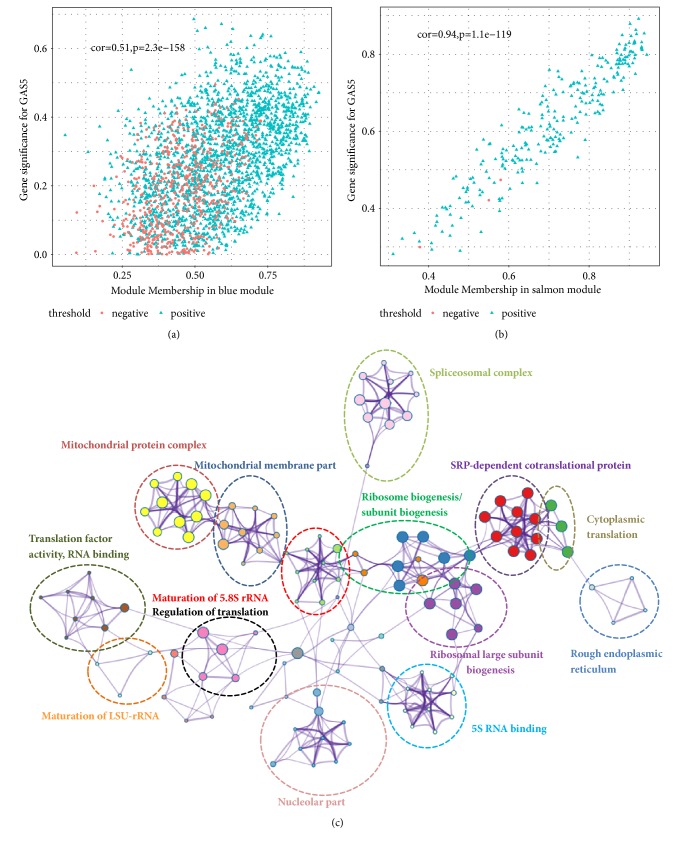
The relationship between gene significance and module membership for the salmon and blue modules. (a-b) Scatterplot of gene significance for GAS5 expression versus module membership for the salmon (a) and blue modules (b). Red indicates a negative correlation with GAS5, and blue indicates a positive correlation with GAS5. (c) The network of gene ontology analysis for biological processes, cellular components, and molecular functions based on genes in the salmon and blue modules.

**Figure 9:**
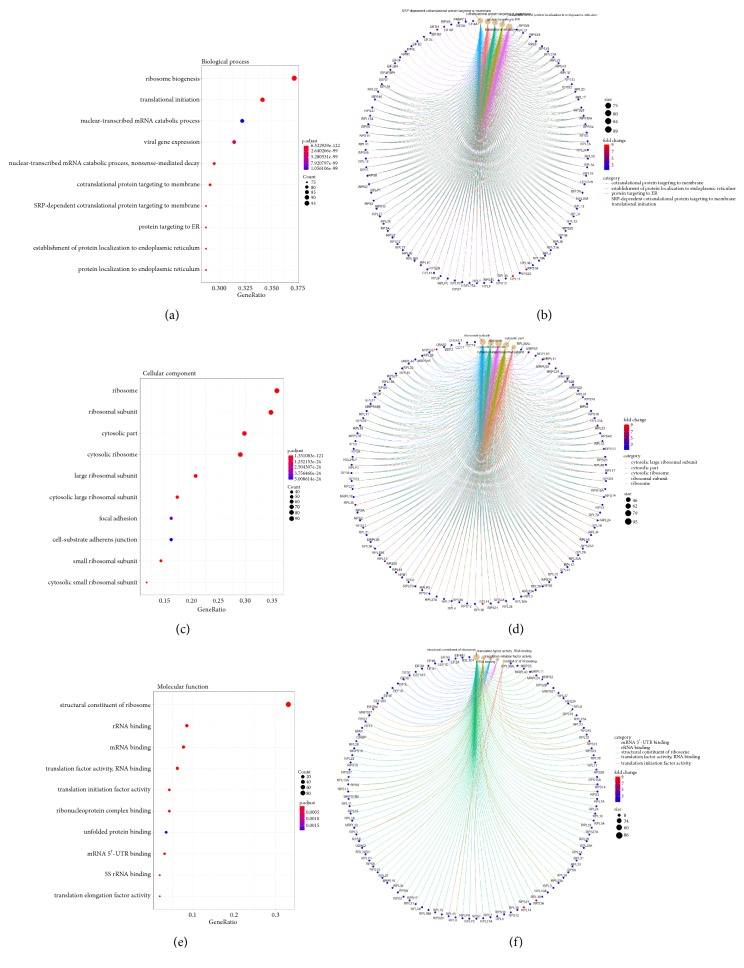
Enrichment analysis of the genes in the salmon and blue modules. (a-b) Gene ontology analysis for biological processes and the gene regulation involved in biological processes. (c-d) Gene ontology analysis for cellular components and the gene regulation involved in cellular components. (e-f) Gene ontology analysis for molecular functions and the gene regulation involved in molecular functions.

**Figure 10:**
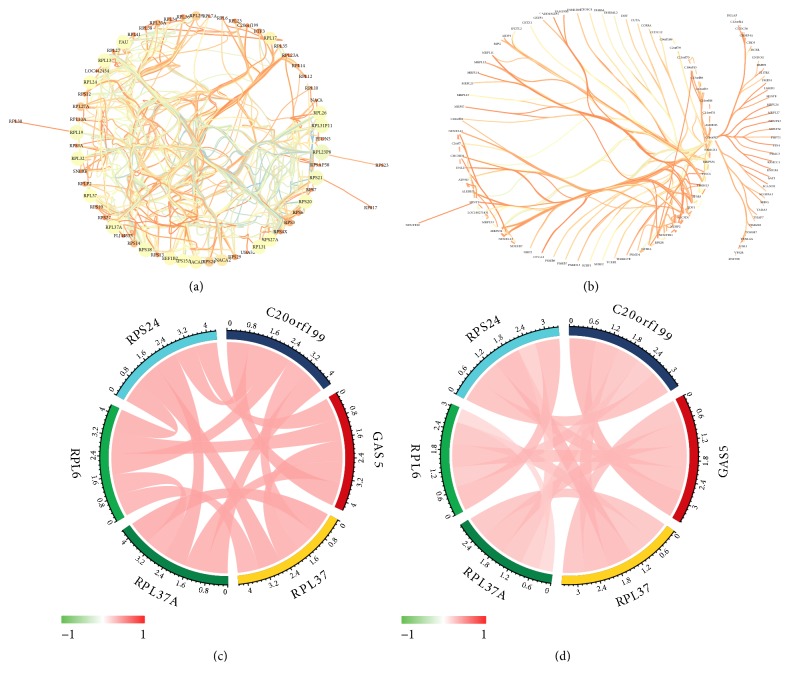
Visualization of genes in the salmon and blue modules with weights higher than the threshold (weight > 0.19) and correlations between GAS5 and the most closely related molecules. Node sizes indicate betweenness centrality, which reflects the amount of control that this node exerts over the interactions of other nodes in the network. Edge sizes depict the weight of the connection between genes. (a) Genes in the salmon module with weights higher than 0.19. (b) Genes in the blue module with weights higher than 0.19. (c) The correlations between GAS5 and the most closely related genes in the TCGA dataset. (d) The validation of the association in the CGGA dataset.

Based on the results above, the salmon and blue modules, which had the most significant relationship with GAS5 expression, were selected. The scatterplot below illustrates genetic significance between GAS5 expression (the correlation of genes with GAS5 expression) and module membership (the correlation of genes with clusters) for the salmon module (Figure 8(a)) and the blue module (Figure 8(b)). The results indicated that genes that had high correlations with the salmon and blue modules were also strongly associated with GAS5 expression. The genes from the salmon and blue modules with coefficients higher than 0.5 were selected and subjected to GO enrichment analysis (Figures 8(c) and 9). rRNA processing, ribonucleoprotein complex biogenesis, translation factor activity, ribosomal subunit biogenesis, and other terms were enriched in the analysis, indicating the key roles of GAS5 in the regulation of ribosomes in LGG tissues (Figures 9(a), 9(c), and 9(e)). The genes involved in GO analysis and their fold changes between the high GAS5 expression group (the 50 samples with the highest GAS5 expression) and the low GAS5 expression group (the 50 samples with the lowest GAS5 expression) are shown on the circular plots (Figures 9(b), 9(d), and 9(f)). RPL18A, RPL23, RPL24, PRL32, PRS12, and other ribosome proteins were highly expressed along with high GAS5 expression, resulting in the upregulation of rRNA processing and ribosome biogenesis. Finally, the network connections in the salmon and blue modules with weight parameters higher than 0.19 from WGCNA were loaded into Cytoscape and analysed. A variety of ribosome proteins, such as RPL10, RPL12, RPL17, and RPS24, were included in the network (Figures 10(a) and 10(b)). High interconnectivity within the gene cluster is depicted by thick edges. The genes most strongly related to GAS5 were analysed in the TCGA dataset and validated with a CGGA (Chinese Glioma Genome Atlas) dataset (Figures 10(c) and 10(d)). The most related genes included RPL6, RPL37A, RPL37, RPS24, and C20orf199, which were included in the salmon module. The results further confirmed the relationship between salmon module and GAS5.

### 3.7. RNA-Seq Analysis of the High and Low GAS5 Expression Groups

**Figure 11:**
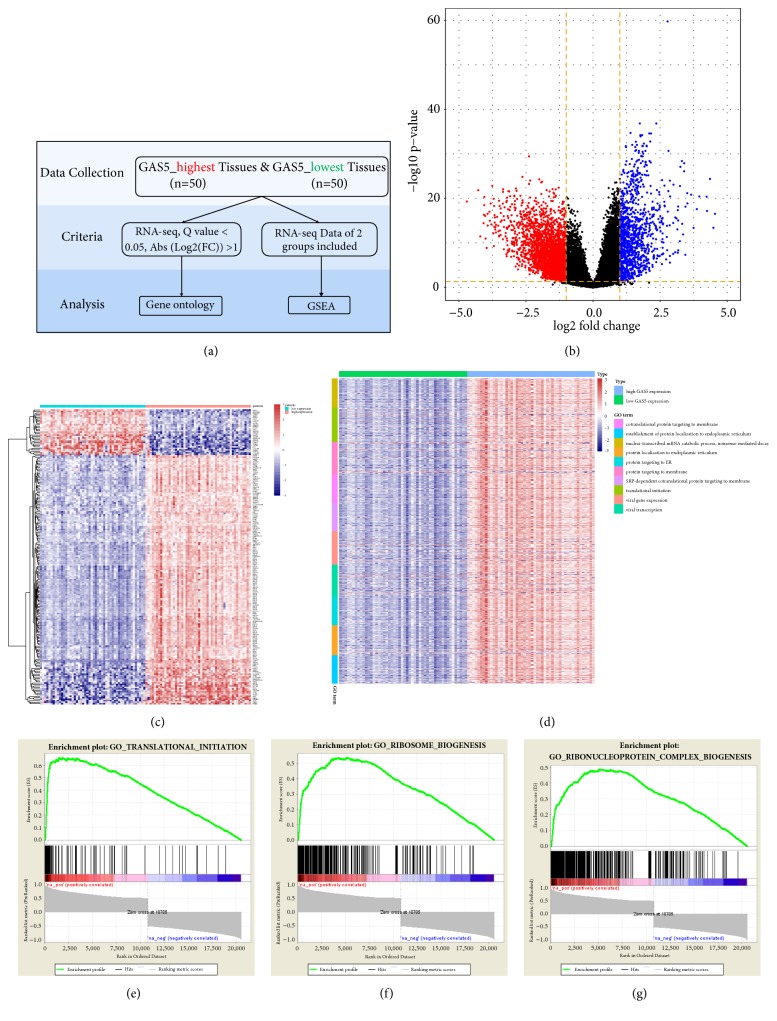
RNA-Seq analysis of LGG tissues with the highest and lowest GAS5 expression. (a) Workflow for RNA-Seq and downstream analysis based on differentially expressed genes and all genes in RNA-Seq. (b) Volcano plot for differentially expressed genes in the groups with high and low expression of GAS5. (c) Heatmap of the 200 differentially expressed genes with the highest significance levels between the groups with high and low expression of GAS5. (d) Heatmap of gene ontology analysis for biological processes. (e-g) Gene set enrichment analysis for the groups with high and low expression of GAS5.

To validate the results of WGCNA, we performed GO analysis and GSEA in the high and low GAS5 expression groups. The workflow is shown in Figure 11(a). The volcano plot indicates the fold changes and Q values of differentially expressed genes (Figure 11(b)). A heatmap of the 200 differentially expressed genes with the highest significance levels between the high and low GAS5 expression groups is shown in Figure 11(c). Then, differentially expressed genes were analysed by GO analysis (Figure 11(d)). The results indicated a high degree of association between GAS5 expression and translation initiation. GSEA was performed using all genes from GAS5 high-expressed group and GAS5 low-expressed group, indicating translation initiation, ribosomal biogenesis, and ribonucleoprotein complex biogenesis were significantly upregulated (Figures 11(e), 11(f), and 11(g)). An MA plot was generated to show the differentially expressed genes with high significance levels between the high and low GAS5 expression groups (Figure S4).

## 4. Discussion

LncRNAs are non-protein-coding transcripts longer than 200 nucleotides and have been demonstrated to play critical roles in diverse cellular processes and the regulation of biological functions [7, 27, 28]. LncRNAs regulate gene expression at the transcriptional, posttranscriptional, and epigenetic levels. The expression levels of lncRNAs are frequently deregulated in cancer [29–32]. For instance, GAS5 has been reported as a crucial tumour suppressor in a variety of human cancers. GAS5 expression is significantly decreased in prostate cancer cells compared with prostate epithelial cells [33]. Ectopic expression of GAS5 induces cell-cycle arrest in the G0–G1 phase by increasing the activity of the P27^Kip1^ promoter. In addition, GAS5 interacts with E2F1 and enhances the binding of E2F1 to the P27^Kip1^ promoter in prostate cancer [33]. GAS5 expression is also markedly downregulated in gastric cancer tissues and influences gastric cancer cell proliferation via regulating the expression of E2F1 and P21 [11]. One study revealed that GAS5 was significantly downregulated in lung adenocarcinoma tissues compared with paired adjacent nontumorous tissue samples. The overexpression of GAS5 varied inversely with the expression of EGFR pathway and IGF-1R proteins in lung adenocarcinoma tissues [12].

Recent studies have implied that lncRNAs participate in the development and malignancy of GBM by regulating glioma stem cell self-renewal, proliferation, differentiation, therapeutic response, and resistance [34–37]. In this study, we focused on GAS5, one crucial noncoding gene in glioma progression [37–39]. GAS5 plays a critical role in the control of mammalian apoptosis and acts as a competitive glucocorticoid response element to mediate cell population growth by suppressing glucocorticoid receptors [9]. GAS5 inhibits the association of glucocorticoid receptors with their DNA recognition sequences by binding to their DNA-binding domain through its double-stranded RNA glucocorticoid receptor element mimic [9]. Thus, GAS5 is upregulated in growth-arrested cells and sensitizes mammalian cells to apoptosis [40]. In this study, we focused on the mRNAs coexpressed with GAS5 in gliomas and how GAS5 controlled the survival of glioma patients. By characterizing the levels of GAS5 expression based on data from TCGA-LGG and TCGA-GBM patients, we demonstrated a significant downregulation of GAS5 expression in LGG and GBM compared with normal brain tissue. One previous study revealed that GAS5 expression decreased as the histologic grade of glioma increased [38]. However, by analysing TCGA-LGG and GBM RNA-Seq datasets, we arrived at the conclusion that histologic grade did not influence GAS5 expression. The discrepancy may be due to the sample numbers. In this study, we analysed the expression levels of GAS5 in 702 samples, far more than Zhao's study used. One previous report revealed that hypermethylation of CpG islands in promoter regions contributes to the downregulation of GAS5 expression [10]. The methylation level of the GAS5 gene was detected by the six probes targeting the CpG island before the transcription start site of GAS5. Both LGG and GBM tissues had low GAS5 methylation levels. However, there was no significant association between the methylation level and the expression level of GAS5. Amplification-type alteration was associated with increased GAS5 expression in both LGG and GBM tissues. GAS5 expression played different roles in affecting the OS of LGG and GBM patients. GAS5 expression was strongly associated with OS in LGG patients. We also examined GAS5 expression in LGG patients with different molecular characteristics and histologic grades. GAS5 expression was higher in IDH1 mutant patients than in patients with wild-type IDH1. To confirm the association between GAS5 and OS in LGG patients, we performed Kaplan-Meier survival analysis on LGG patients according to their GAS5 expression levels, stratified by clinicopathological characteristics. Kaplan-Meier survival analysis showed high association between GAS5 expression level and OS in both grade II and grade III gliomas. We also performed multivariate survival analysis of GAS5 with clinical and molecular characteristics in LGG patients. The Cox HR model indicated that GAS5 was strongly associated with the overall survival of patients. Based on the results discussed above, a nomogram was developed to predict the 3-year and 5-year survival probabilities of LGG patients. Time-dependent ROCs and log-rank tests further confirmed the results.

To understand the molecular mechanism behind the effect of GAS5 in LGG tissues, we performed WGCNA and downstream enrichment analysis. The results indicated that the genes in the salmon and blue modules played important roles in ribosome function, which was consistent with previous reports on the involvement of GAS5 in ribosomal RNA biosynthesis [41]. In addition, the genes highly correlated with GAS5 expression showed intensive connection when visualized by Cytoscape. A variety of the hub genes were ribosomal proteins, e.g., RPL13, RPL37, PRL38, and PRL10A, indicating the strong association between GAS5 and ribosomal function. The results were consistent with a previous study, which revealed that GAS5 may act as a “ribo-repressor” of glucocorticoid receptors to influence cell survival and metabolic activity during starvation by modulating the transcriptional activity of the glucocorticoid receptor [40]. To further confirm the results from WGCNA, we also performed RNA-Seq analysis and downstream GO analysis and GSEA. Translation initiation was the most predominant result from GO, implying that GAS5 may be an important regulator of transcription initiation. One study demonstrated that GAS5 was enriched with eIF4E, which had two RNA binding motifs for GAS5 [42]. The deletion of either motif inhibited the binding of GAS5 to eIF4E. In addition, GSEA results also indicated upregulation of translation initiation and ribosomal biogenesis in group with high GAS5 expression, which was consistent with GO analysis and previous research [43].

Altogether, the data presented here suggest that GAS5 plays essential roles in the physiological process of LGG. The mechanism may include ribosome biogenesis and regulation of translation initiation. In conclusion, based on molecular and clinical analysis, our study demonstrated that GAS5 is linked tightly to ribosomal biogenesis and translation initiation in LGG tissues and acts as a predictor of survival in patients with early-stage gliomas.

## Figures and Tables

**Table 1:** The associations between GAS5 expression and pathological parameters in patients.

**Attributes** GAS expression and …	**GBM**			**LGG**		
	**Statistic**	**P-value**	**FDR (BH)**	**Statistic**	**P-value**	**FDR (BH)**
Ethnicity (Wilcox test)	0.096	0.620	0.843	0.002	0.487	0.487

Histological type (Kruskal-Wallis test)	2.467	0.291	0.843	3.918	0.141	0.197

Race (Kruskal-Wallis test)	1.110	0.574	0.843	4.180	0.243	0.283

Radiation therapy (Wilcox test)	-0.003	0.843	0.843	-0.025	0.019	0.045

Tumour purity (Spearman correlation)	-0.023	0.785	0.843	0.300	1.775e-11	1.243e-10

Age (Spearman correlation)	-0.064	0.446	0.843	-0.093	0.041	0.073

## Data Availability

The data used to support the findings of this study are available from the corresponding author upon request.

## Supplementary Materials

Supplementary Materials Figure S1: the GAS5 expression in different stages of glioma (a) and the correlation of GAS5 methylation level and GAS5 expression level in GBM (b) and LGG (c) tissues. Figure S2: the associations between GAS5 expression level and tumour purity in LGG (a) and GBM (b) patients. Figure S3: the dendrogram of modules identified by WGCNA. Figure S4: MA plot of differentially expressed genes with high significance levels in the groups with high and low GAS5 expression groups in LGG tissues.
