# Supplementary material for: Low GAS5 Levels as a Predictor of Poor Survival in Patients with Lower-Grade Gliomas
Source: J Oncol. 2019 Feb 3;2019:1785042. doi: 10.1155/2019/1785042 (PMC6377997; doi:10.1155/2019/1785042)
Supplement: Supplementary Materials — Figure S1: the GAS5 expression in different stages of glioma (a) and the correlation of GAS5 methylation level and GAS5 expression level in GBM (b) and LGG (c) tissues. Figure S2: the associations between GAS5 expression level and tumour purity in LGG (a) and GBM (b) patients. Figure S3: the dendrogram of modules identified by WGCNA. Figure S4: MA plot of differentially expressed genes with high significance levels in the groups with high and low GAS5 expression groups in LGG tissues. [file 1785042.f1.pdf]

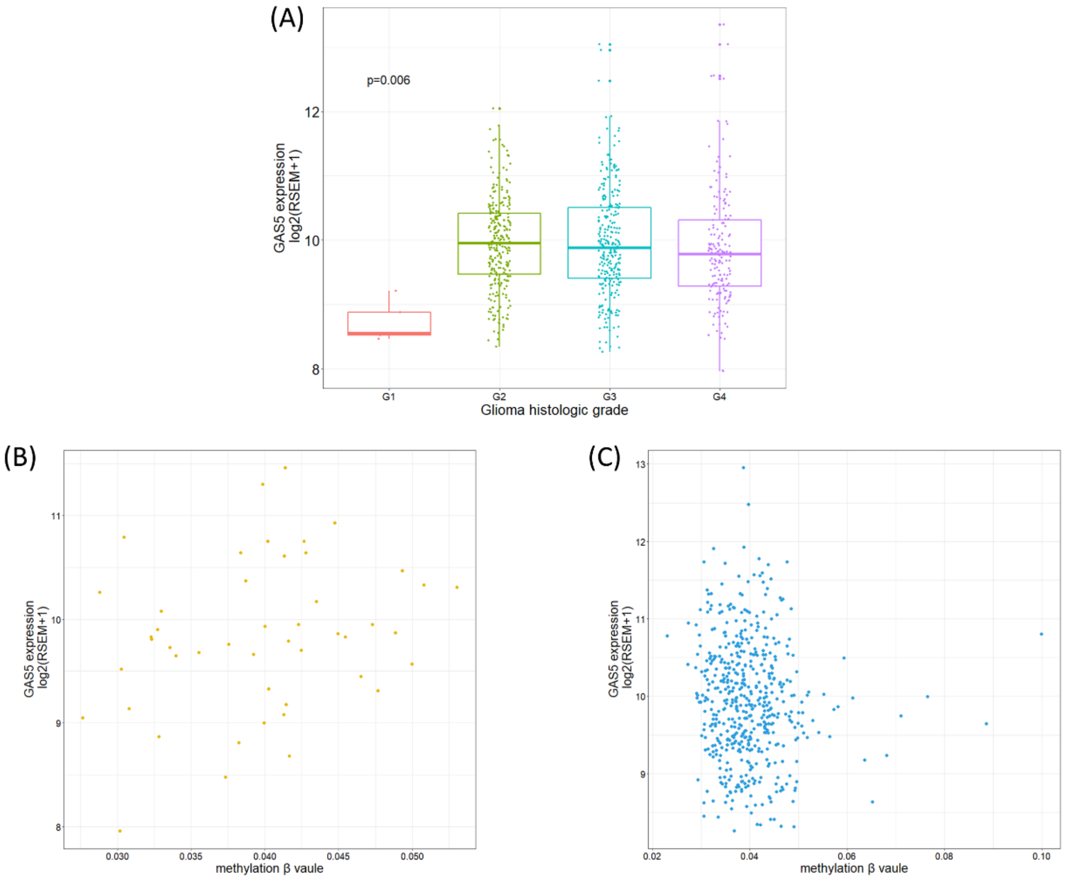

Figure. S1

The GAS5 expression in different stages of glioma (a) and the correlation of GAS5 methylation level and GAS5 expression level in GBM (b) and LGG (c) tissues.

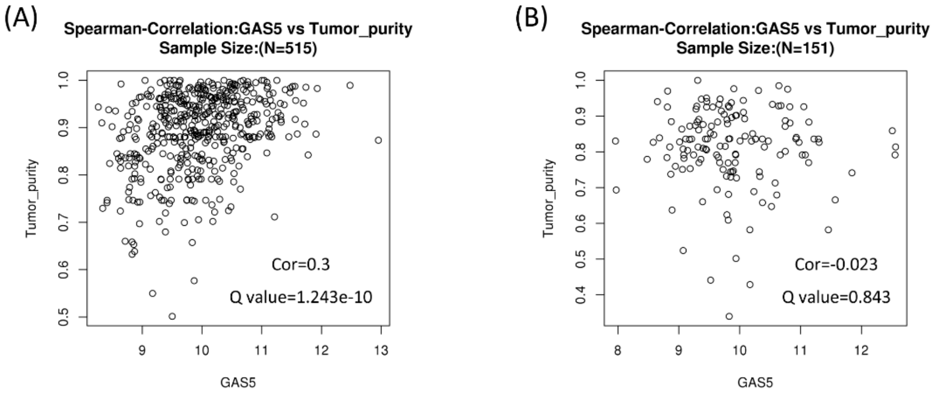

Figure. S2

The associations between GAS5 expression level and tumor purity in LGG (a) and GBM (b) patients.

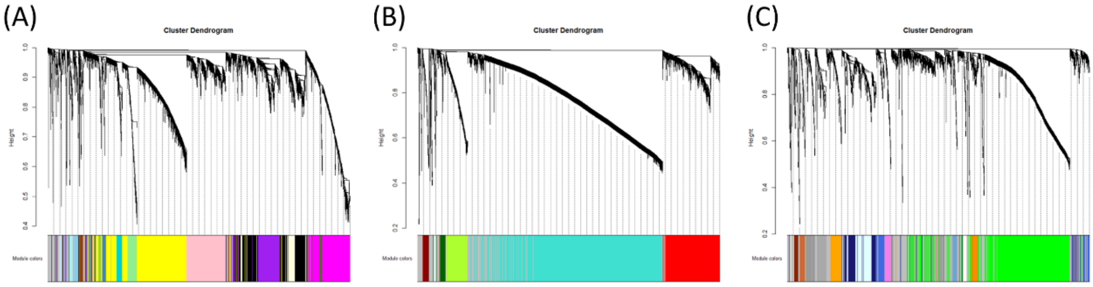

Figure. S3

The dendrogram of modules identified by WGCNA.

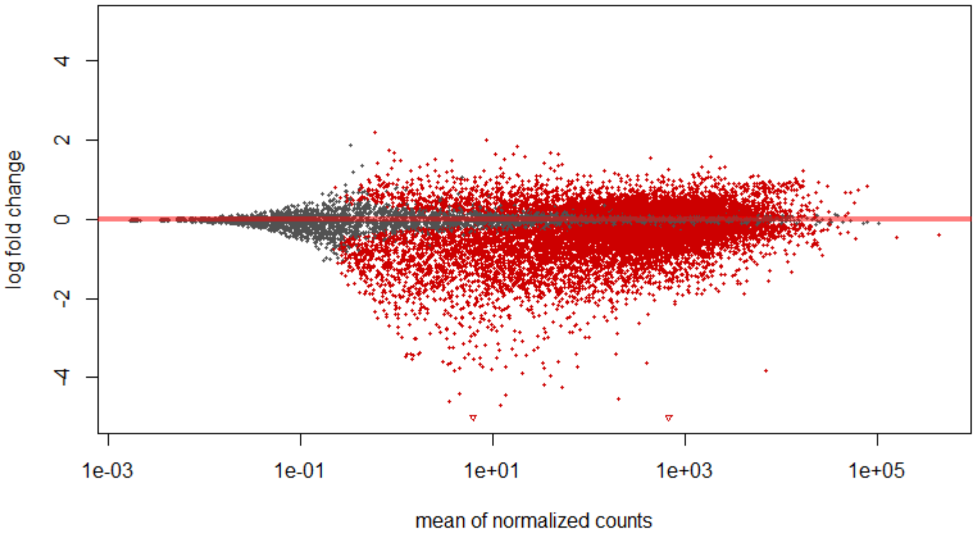

Figure. S4

The MAplot of significant differentially expressed genes in high GAS5 expression and low GAS5 expression groups from LGG tissues.
